# Accompanying Symptoms Overlap during Attacks in Menière’s Disease and Vestibular Migraine

**DOI:** 10.3389/fneur.2014.00265

**Published:** 2014-12-15

**Authors:** Jose Antonio Lopez-Escamez, Julia Dlugaiczyk, Julien Jacobs, Thomas Lempert, Roberto Teggi, Michael von Brevern, Alexandre Bisdorff

**Affiliations:** ^1^Otology and Neurotology Group CTS495, Department of Genomic Medicine, Centre for Genomics and Oncology Research, Pfizer/Universidad de Granada/Junta de Andalucía (GENyO), Granada, Spain; ^2^Department of Otolaryngology, Hospital de Poniente, El Ejido, Almeria, Spain; ^3^Department of Otorhinolaryngology, Saarland University Medical Center, Homburg, Germany; ^4^Public Research Centre for Health (CRP-Santé), Center for Health Studies, Luxembourg, Luxembourg; ^5^Department of Neurology, Schlosspark-klinik, Berlin, Germany; ^6^ENT Division, San Raffaele Scientific Institute, Milan, Italy; ^7^Department of Neurology, Park-Klinik Weissensee, Berlin, Germany; ^8^Department of Neurology, Centre Hospitalier Emile Mayrisch, Esch-sur-Alzette, Luxembourg

**Keywords:** vertigo, migraine, sensorineural hearing loss, clinical diagnosis, vestibular disorders, headache

## Abstract

Menière’s disease and vestibular migraine (VM) are the most common causes of spontaneous recurrent vertigo. The current diagnostic criteria for the two disorders are mainly based on patients’ symptoms, and no biological marker is available. When applying these criteria, an overlap of the two disorders is occasionally observed in clinical practice. Therefore, the present prospective multicenter study aimed to identify accompanying symptoms that may help to differentiate between MD, VM, and probable vestibular migraine (pVM). Two hundred and sixty-eight patients were included in the study (MD: *n* = 119, VM: *n* = 84, pVM: *n* = 65). Patients with MD suffered mainly from accompanying auditory symptoms (tinnitus, fullness of ear, and hearing loss), while accompanying migraine symptoms (migraine-type headache, photo-/phonophobia, visual aura), anxiety, and palpitations were more common during attacks of VM. However, it has to be noted that a subset of MD patients also experienced (migraine-type) headache during the attacks. On the other hand, some VM/pVM patients reported accompanying auditory symptoms. The female/male ratio was statistically higher in VM/pVM as compared to MD, while the age of onset was significantly lower in the former two. The frequency of migraine-type headache was significantly higher in VM as compared to both pVM and MD. Accompanying headache of any type was observed in declining order in VM, pVM, and MD. In conclusion, the present study confirms a considerable overlap of symptoms in MD, VM, and pVM. In particular, we could not identify any highly specific symptom for one of the three entities. It is rather the combination of symptoms that should guide diagnostic reasoning. The identification of common symptom patterns in VM and MD may help to refine future diagnostic criteria for the two disorders.

## Introduction

The most common causes of spontaneous recurrent vertigo are vestibular migraine (VM) and Menière’s disease (MD) (1). In the absence of a diagnostic gold standard and confirmatory tests, the diagnosis is largely based on the history, including the duration of vestibular symptoms, accompanying cochlear and neurologic symptoms and exclusion of other causes (2, 3). Diagnostic criteria for VM have recently been published by the Bárány Society and the International Headache Society (2, 4). A previous version of these operational criteria has shown high reliability and validity (5). The criteria for VM require that in a person with a past or ongoing history of migraine, at least 50% of the vestibular episodes are accompanied by migrainous symptoms such as headache, visual aura, and/or photo- and phonophobia (2, 4). For probable vestibular migraine (pVM), either a history of migraine or migraine symptoms during the attack are sufficient (2, 4). The criteria for MD by the American Academy of Otolaryngology-Head and Neck Surgery (AAO-HNS) require accompanying auditory symptoms and audiometrically documented hearing loss on at least one occasion (6). Vestibular testing results are neither required for a diagnosis of VM nor for MD.

Despite these operational criteria, the interface between vertigo and headache is complex (7) and the discrimination between MD and VM remains a challenge, in particular in the early course of the disorders (8–11). Of note, patients with MD have a disproportionally high prevalence of headache, including migraine (12–14). Furthermore, cochlear symptoms are not exclusive to MD but may similarly occur during attacks of VM (15, 16). Only patients who have two different types of attacks, one fulfilling the criteria for VM and the other for MD, should be diagnosed with both disorders (4).

The development of internationally accepted diagnostic criteria is quite recent in the vestibular field (2, 4). At an early stage, the development of disease classifications and of diagnostic criteria is mainly based on expert opinions rather than on robust data (17). Once established, these criteria can be widely applied and their validity can be tested. One way of validation is to assess the stability of the diagnosis over time (18).

The aim of this study is to systematically assess accompanying symptoms of vestibular attacks in patients fulfilling current criteria for VM, pVM, and MD to enhance the phenotypic description of these episodic vestibular disorders. As a result, the features, which discriminate best or least between the two, should become clearer and help to guide the clinician in his history taking and diagnostic decisions. The data could also contribute to the discussion if VM and MD are clearly different entities or rather part of a clinical spectrum.

## Patients and Methods

### Patients

Four hundred and twenty-three patients presenting with an episodic vestibular syndrome were recruited between August 2013 and March 2014 in a prospective multicenter study performed in Italy, Luxembourg, Germany and Spain in six clinical centers. All patients were interviewed by experienced neuro-otologists (three neurologists and three otolaryngologists) with at least 12 years of clinical practice. The centers were tertiary referral outpatient clinics or vertigo clinics in general hospitals. For the present analysis, we selected a total of 268 patients fulfilling either the diagnostic criteria for MD according to the American Academy of Head and Neck Surgery (6) or the diagnostic criteria for VM or pVM (2, 4) to characterize the clinical symptoms during the episodes of vertigo. Patients fulfilling the criteria for both MD and VM (either VM or pVM) were excluded from the study.

This study was approved by the local ethics committees of all participating centers. All patients gave written informed consent before entering the study.

### Methods

All patients were examined by clinical testing for spontaneous and positional nystagmus, smooth pursuit, saccades, and head impulse test. A pure tone audiogram was performed in all patients to determine bone and air conduction hearing thresholds. Additional tests to exclude other differential diagnoses were performed at the discretion of each clinician to establish the diagnosis.

A structured questionnaire was designed to record the symptoms according to the Bárány Vestibular Symptoms Grid to characterize the vestibular phenotype (19). The questionnaire was designed to collect all vestibular symptoms reported by patients (vertigo, dizziness, vestibulo-visual symptoms, postural symptoms), the frequency, and duration of the attacks and the intensity of the symptoms. The questionnaire also included basic demographic data (age and gender), patient’s age at onset of vestibular symptoms, and a set of questions to determine the accompanying symptoms occurring during the attacks, i.e., vision-related symptoms (photophonia, visual aura, diplopia), hearing-related symptoms (phonophobia, tinnitus, fullness of ear, hearing loss), vegetative symptoms (nausea, vomiting, palpitations, choking), emotional symptoms (anxiety), and headache (Table 1). Patients were able to choose if accompanying symptoms occurred never, sometimes (<50% of attacks) or mostly (≥50% of attacks).

**Table 1:** **Summary of the structured questionnaire designed to characterize symptoms in the vestibular episodic syndrome**.

Questions		
Socio-demographic	Gender, date of birth	
Duration of vestibular syndrome		
Symptoms quality (according to the Bárány Vestibular Symptoms grid)	Vertigo, dizziness, vestibule-visual symptoms, postural symptoms	
Attack frequency	Frequency	
	Clusters^a^	Usual duration
	Residual symptoms between attacks	Symptoms
		During clusters?
Attack duration	Core event	
	Duration of recovery time	
	Distinctive exacerbation?	Duration
		Frequency
Intensity of symptoms	Kind of attack (mild, moderate, severe)	
Accompanying symptoms of attacks	Vision related	Photophobia
		Visual aura
		Diplopia
	Hearing related	Phonophobia
		Tinnitus
		Fullness of ear
		Hearing loss
	Vegetative	Nausea
		Vomiting
		Palpitations
		Choking
	Emotional	Anxiety
	Headache	Unilateral
		Pulsating quality
		Worse on effort
		Moderate or severe intensity
Clinical diagnosis		

*^a^Clusters refer to the temporal aggregation in episodes of vestibular symptoms*.

To characterize the type of headache during the attack, the interview included specific questions concerning localization (unilateral?), quality (pulsating ?), intensity of headache (moderate to severe?), and aggravation of headache by activity. Each question could be answered as never, sometimes or mostly and scored as 0, 1, or 2, respectively. A composite migraine risk score (MRS) was calculated for the patients suffering from headache as the sum of these four items. Thus, MRS ranged from 0 to a maximum of 8.

A statistical analysis was performed using SAS 9.3 software. Absolute and relative frequencies were calculated for each accompanying symptom occurring during the attacks. The observed frequency of each symptom was compared between patients with MD, VM, and pVM using Chi^2^ test with Yates’ correction or Fisher’s exact test. Student’s *t* tests were used to compare the age of onset of pVM, VM, and MD. A *p*-value <0.05 was considered statistically significant.

## Results

**Figure 1:**
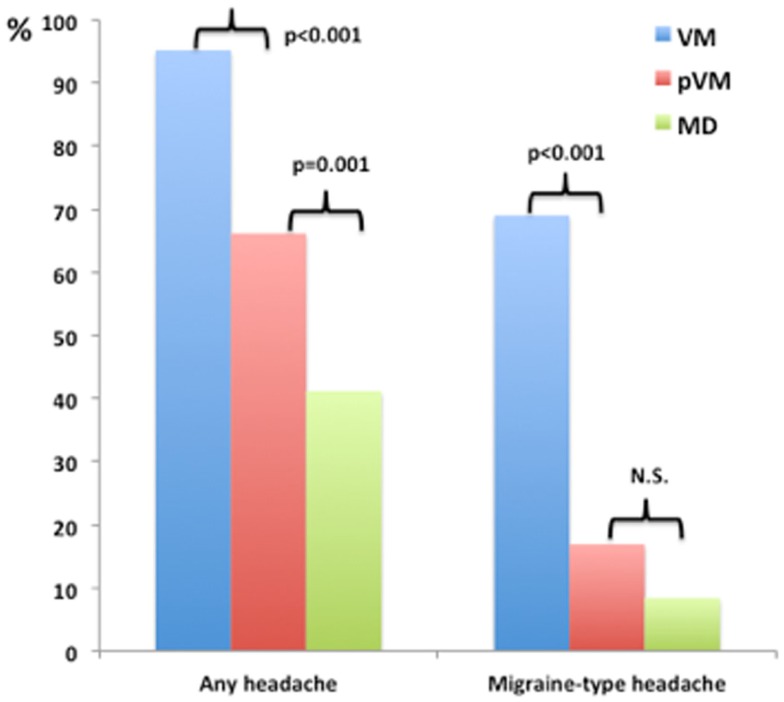
**Relative frequency of headache and migraine-type headache during the episode of vestibular symptoms among patients with Menière’s disease (MD), vestibular migraine (VM), and probable VM (pVM)**.

One hundred and nineteen patients with MD were included in the study. The age at onset of vertigo was 48 ± 13 years and the female/male ratio was 1.2. The accompanying symptoms reported during the episodes of vertigo are shown in Table 2. The most common symptoms in patients with MD (total frequency) were nausea (94%), vomiting (84%), and tinnitus (83%). Accompanying hearing loss was reported in 77% of cases, headache was found in 41% of patients with MD, and migraine-type headache was observed in 8.4% of MD patients during the attack (Figure 1). Although we did not study the relationship between perceived hearing loss and hearing threshold in the audiogram systematically, we noted that some patients with MD, who did not report hearing loss during attacks, had a profound chronic sensorineural hearing loss on the audiogram (>70 dB normal hearing level).

**Table 2:** **Accompanying symptoms reported by patients with definite and probable VM during the attack**.

Symptoms	Relative frequency (%)					
	MD		VM		Probable VM	
	Mostly	Sometimes	Mostly	Sometimes	Mostly	Sometimes
Nausea	80.7	13.4	76.2	17.9	43.1	43.1
Tinnitus	68.1	15.1	20.2	26.2	9.2	21.5
Hearing loss	61.3	16.0	10.7	15.5	9.2	6.2
Fullness of ear	61.3	19.3	14.3	20.2	7.7	20.0
Phonophobia	31.1	31.1	60.7	19.1	32.3	29.2
Photophobia	21.0	20.2	57.1	22.6	26.1	33.9
Visual aura	0.8	10.1	13.1	19.0	6.2	9.2
Anxiety	34.4	43.7	50.0	40.5	44.6	35.4
Vomiting	46.2	37.8	23.8	45.2	7.7	20.0
Palpitations	3.4	31.1	14.3	35.7	13.9	41.5
Choking	5.0	6.7	7.1	15.5	7.7	10.8
Diplopia	0.0	7.6	2.4	9.5	1.5	6.2
Headache	41.2		95.2		66.1	
Migraine-type headache	8.4		69.1		16.9	
Headache features
Worse on effort	20.4	40.8	62.5	23.8	27.9	51.2
Moderate of severe	20.4	40.8	57.5	35.0	18.6	53.5
Unilateral	10.2	40.8	52.5	27.5	14.0	48.8
Pulsating quality	18.4	38.8	45.0	35.0	20.9	48.8

Another 84 patients had VM and 65 had PVM. The age at onset of the disease was 43 ± 14 years for VM and 42 ± 13 years for pVM, being both significantly lower when compared with MD (*t* test, *p* = 0.007 for VM and *p* = 0.005 for pVM). The female/male ratio was 7.4 and 4.4, respectively. The most frequent accompanying symptom in both groups was headache (95% in VM and 66% in pVM), followed by nausea, anxiety, phonophobia, and photophobia (Table 2). Comparing the frequency of symptoms between VM and pVM, we found that the following symptoms were more common in patients with VM: photophobia, vomiting, and headache (all *p* < 0.001) and also phonophobia (*p* = 0.002). Moreover, headache with migrainous features was observed more frequently in VM than in pVM patients (unilateral, *p* < 0.001; moderate or severe intensity, *p* < 0.001; pulsating quality, *p* = 0.03; worse on effort, *p* = 0.001).

Comparing the frequency of symptoms between MD and VM, we found that tinnitus, aural fullness, and hearing loss were less common in patients with VM than MD (all, *p* < 0.001). Moreover, vomiting was also most frequently observed in patients with MD than VM (*p* = 0.002). Conversely, photo- and phonophobia (*p* < 0.001), visual aura (*p* < 0.001), palpitations (*p* = 0.008), and anxiety (*p* = 0.024) were reported more often by patients with VM than MD.

The percentage of patients with VM, pVM, and MD reporting headache during vestibular attacks was 95.2, 66.1, and 41.2% for, respectively. Among patients reporting headache, the headache compatible with migraine (headache with at least two of the four features mentioned above during most of the episodes) was found in 72.5, 25.6, and 20.4% for VM, pVM, and MD. The frequency of headache (any type) and migraine-type headache was significantly higher in patients with VM compared to patients with pVM and MD (Figure 1; *p* < 0.001 each). The MRS was also significantly higher in patients with VM compared to either pVM or MD (*p* < 0.001 each; MRS was 5.6 ± 1.8 in VM, 3.7 ± 1.8 in pVM, and 3.0 ± 2.1 in MD), but interestingly no significant difference was observed between patients with MD and pVM. Of note, no patient in our series with a diagnosis of MD or pVM had a MRS = 8, and only patients with a diagnosis of VM reached the maximum score.

## Discussion

This study demonstrates, quite expectedly, that patients with VM and pVM have mostly migraine symptoms during their attacks while patients with MD have mostly auditory symptoms. On the other hand, there is a considerable overlap of accompanying symptoms. Our findings suggest that both VM and pVM are more commonly found in women (four to eight times), which does not apply to MD. Migraine is three times more common in women and it typically occurs in young patients with a peak prevalence in the fourth decade, regardless of the presence of vestibular symptoms (20).

Moreover, the mean age of onset in our cohorts is different between VM/pVM and MD with VM/pVM manifesting earlier. Of note, a relevant subset of patients with MD, around 40%, presents with headaches during their vertigo attacks and, interestingly, more than 8.4% of patients with MD describe headaches compatible with migraine (i.e., 20% of MD patients suffering from headache describe headaches compatible with migraine). In this study, we excluded patients fulfilling the criteria for both MD and VM, so we are probably underestimating the prevalence of headache, including migraine, and hearing loss in our series compared to an unselected patient population presenting with episodic vertigo. Neither headache nor migraine is considered as an exclusion criterion in MD in the current definition of the AAO-HNS (6), nor do the instructions warn the clinician to carefully consider VM as a differential diagnosis. In fact, the presence of headache, including migraine, during the vertigo attack does not rule out the diagnosis of MD (7, 12). Phonophobia and photophobia were also reported by some patients with MD, and these concurrent symptoms can make the differentiation between MD and VM/pVM confusing as these symptoms are part of the VM/pVM criteria (2, 4).

A second finding of our study is that a subset of patients with VM also experience auditory symptoms, especially tinnitus, during the attack. The definition of VM requires that at least 50% of the attacks are accompanied by migraine features (2, 4), but auditory symptoms are not considered except phonophobia. This also suggests an overlapping phenotype between patients with VM and MD. However, our results indicate that lack of certain auditory symptoms, especially hearing loss, are suggestive for a diagnosis of either VM or pVM. Phonophobia is similarly frequent in MD and pVM, but more frequent in VM.

A third point to mention from our data is that anxiety and palpitations and nausea are more common symptoms during the attack in patients with VM compared with MD. Vomiting occurs in most patients with MD and it has a lower frequency. In VM, but it is only found in one out of four patients with pVM. Vomiting may be part of migraine as such, as well as a consequence of vertigo, vomiting in association with auditory symptoms may help to discriminate between MD and VM.

A subset of patients with MD, who showed hearing loss in the audiogram after the episode, did not perceive hearing loss during the attack. This observation applies especially to patients with a constant severe sensorineural hearing loss on the affected ear, which temporarily got worse during the vertigo attack. This shows that hearing loss can be missed by history taking and underlines the importance of audiometric testing in all patients with episodic vertigo to prevent a delay in the diagnosis of MD.

The MRS is a composite score aiming to estimate the probability that a headache has a migrainous character based on four questions. However, this score does not consider the number of headache attacks and it cannot replace the IHS criteria for the diagnosis of migraine (4). Our data demonstrate that the MRS can be useful to differentiate MD from VM, since VM patients reached a higher score than patients with MD or pVM. This is likely to be, at least in part, a direct result of the diagnostic criteria. Nevertheless, a high MRS score may point to VM, in particular when hearing loss is absent since only patients with VM reached the maximum score (MRS = 8). Although this finding should be validated in a larger cohort of patients with MD, VM, and pVM, the application of the MRS in clinical practice to improve the diagnostic accuracy of VM seems promising.

Our study has several limitations. Anxiety and depression are comorbid conditions with MD and VM, and we did not use a specific instrument for their diagnosis since this study was not designed to estimate the comorbidities of these psychiatric conditions. Psychiatric comorbidity has recently been reported in half of patients with vertigo/dizziness using a structured clinical interview for major mental disorders (21). A second limitation is the fact that the family history of migraine or MD was not considered for the diagnosis and both VM and MD have a strong trend for familial clustering (22). Since there is a frequent association between episodic vertigo, migraine, and MD in close relatives, including identical twins, it is possible that MD and VM can share a common genetic link with variable expression of hearing loss, episodic vertigo, visual aura, and migraine in the same families (23). Finally, for the diagnosis of definite VM, the diagnosis of migraine was made according to IHS criteria, but regarding the quality of the headache accompanying the vertigo episodes, our questionnaire only allowed to establish how many migraine features were present, but not if it fulfilled a migraine diagnosis according to IHS criteria.

All of the accompanying symptoms studied here (except visual aura) may occur in these three disorders but with different frequencies. In summary, subjective hearing loss, tinnitus, and fullness of the ear are much more common in MD. We did not assess severity of symptoms, which may further aid the differentiation of VM and MD. According to our clinical experience, we would expect that hearing loss or tinnitus during attacks of MD were more pronounced than during attacks of VM, which needs to be investigated in further studies. Visual aura, if present, points toward VM. Although both, photophobia and phonophobia, are more frequent in VM than in MD, they do not seem to have strong discriminating power between pVM and MD, while headache performs slightly better and favors a diagnosis of VM. It is somewhat discomforting that some essential criteria of the VM definition – the accompanying migraine symptoms – are relatively weak discriminators against MD. Instead, it is the absence or presence of hearing symptoms (except phonophobia) and the absence/presence of hearing loss on audiometry, which really count. It is likely, although unproven, that migraine symptoms during the attack are more useful for distinction of VM from other causes of recurrent vertigo, such as benign paroxysmal positional vertigo, vertebrobasilar TIA, or vestibular paroxysmia.

In conclusion, the present study shows that differences in accompanying symptoms during attacks in MD, VM, and pVM are present in a group comparison, but the considerable overlap of symptoms renders the differential diagnosis in the individual patient challenging. Future studies may address the question whether VM and MD just overlap with regard to clinical phenomenology or if they represent variant phenotypes of a broad clinical spectrum disorder.

## Conflict of Interest Statement

The authors declare that the research was conducted in the absence of any commercial or financial relationships that could be construed as a potential conflict of interest.
